# Morphology-Aware Deep Features and Frozen Filters for Surgical Instrument Segmentation with LLM-Based Scene Summarization

**DOI:** 10.3390/jcm15062227

**Published:** 2026-03-15

**Authors:** Adnan Haider, Muhammad Arsalan, Kyungeun Cho

**Affiliations:** 1 Department of Computer Science and Artificial Intelligence, College of Advanced Convergence Engineering, Dongguk University-Seoul, 30 Pildongro 1-gil, Jung-gu, Seoul 04620, Republic of Korea; adnanhaider@dgu.ac.kr; 2 College of Engineering, Qatar University, Doha 2713, Qatar; muhammad.arsalan@qu.edu.qa

**Keywords:** artificial intelligence, medical image analysis, semantic segmentation, surgical instrument, robotic surgery, LLM

## Abstract

**Background/Objectives:** The rise of artificial intelligence is injecting intelligence into the healthcare sector, including surgery. Vision-based intelligent systems that assist surgical procedures can significantly increase productivity, safety, and effectiveness during surgery. Surgical instruments are central components of any surgical intervention, yet detecting and locating them during live surgeries remains challenging due to adverse imaging conditions such as blood occlusion, smoke, blur, glare, low-contrast, instrument scale variation, and other artifacts. **Methods**: To address these challenges, we developed an advanced segmentation architecture termed the frozen-filters-based morphology-aware segmentation network (FFMS-Net). Accurate surgical instrument segmentation strongly depends on edge and morphology information; however, in conventional neural networks, this spatial information is progressively degraded during spatial processing. FFMS-Net introduces a frozen and learnable feature pipeline (FLFP) that simultaneously exploits frozen edge representations and learnable features. Within FLFP, Sobel and Laplacian filters are frozen to preserve edge and orientation information, which is subsequently fused with learnable initial spatial features. Moreover, a tri-atrous blending (TAB) block is employed at the end of the encoder to fuse multi-receptive-field-based contextual information, preserving instrument morphology and improving robustness under challenging conditions such as blur, blood occlusion, and smoke. Datasets focused on surgical instruments often suffer from severe class imbalance and poor instrument visibility. To mitigate these issues, FFMS-Net incorporates a progressively structure-preserving decoder (PSPD) that aggregates dilated and standard spatial information after each upsampling stage to maintain class structure. Multi-scale spatial features from different encoder levels are further fused using light skip paths (LSPs) to project channels with task-relevant patterns. **Results/Conclusions:** FFMS-Net is extensively evaluated on three challenging datasets: UW-Sinus-surgery-live, UW-Sinus-cadaveric, and CholecSeg8k. The proposed method demonstrates promising performance compared with state-of-the-art approaches while requiring only 1.5 million trainable parameters. In addition, an open-source large language model is integrated for non-clinical summarization of the surgical scene based on the predicted mask and deterministic descriptors derived from it.

## 1. Introduction

Surgical interventions are prevalent globally. Minimally invasive surgeries are preferred over open surgeries because they involve smaller incisions and enable faster recovery, wherever applicable [1]. Endoscopic and laparoscopic procedures are common examples of minimally invasive surgery. In these procedures, surgical instruments, along with a camera, are inserted through natural openings of the human body [2]. Endoscopic sinus surgery and laparoscopic gallbladder removal are widely practiced examples [3]. Despite the advantages of endoscopic surgical interventions, several limitations remain, including a restricted field of view and challenging visual conditions during live surgery [4]. In this context, detecting and locating surgical instruments is critically important yet remains challenging. During live surgery, factors such as blur, blood obscuration, size variability, smoke, low contrast, glare, complex structure, and other artifacts complicate accurate pixel-level instrument detection [5]. Addressing these challenges requires a comprehensive framework capable of precise surgical instrument detection at the pixel level.

**Figure 1:**
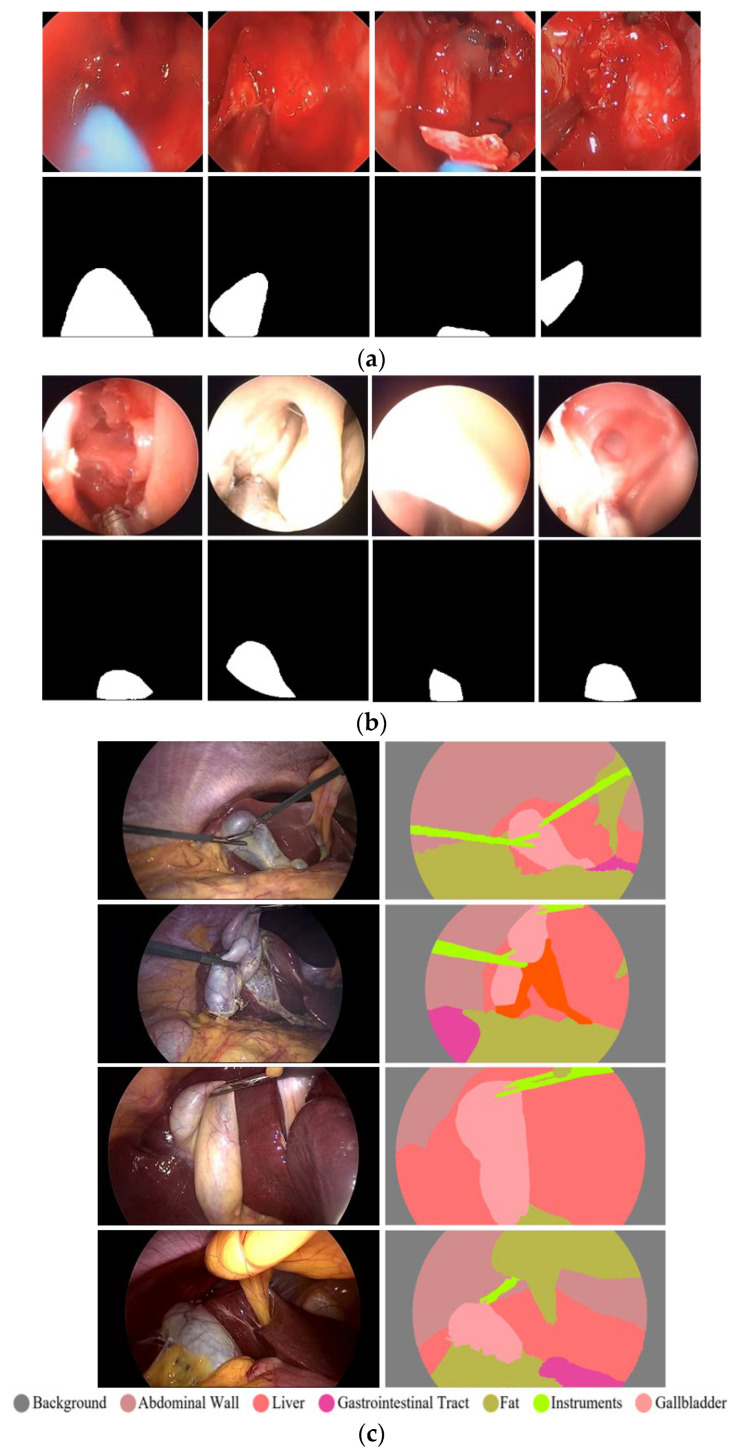
Example images of the datasets along with their corresponding ground truth images. (**a**) UW-Sinus-surgery-live, (**b**) UW-Sinus-cadaveric, and (**c**) CholecSeg8K.

Accurate segmentation of surgical instrument boundaries is equally important and even more challenging under the aforementioned conditions. Surgical instrument segmentation is a key component of robot-assisted minimally invasive surgery, where precise spatial localization directly impacts procedural safety and effectiveness. Accordingly, we developed an advanced segmentation model designed to deliver robust performance under challenging imaging conditions. The proposed model is evaluated using three publicly available and diverse datasets. Sample images from these datasets are presented in Figure 1. Specifically, this study uses UW-Sinus-surgery-live [6], UW-Sinus-cadaveric [6], and choelcSeg8k [7]. As illustrated by the sample images, all three datasets exhibit substantial inter- and intra-dataset variability. In Figure 1a, example images from the UW-Sinus-surgery-live dataset are shown. This dataset was created from live sinus surgery, and a substantial portion presents serious challenges for accurate surgical instrument segmentation. Sample images from UW-Sinus-surgery-live include blurred, blood-occluded, small-scale, and low-visibility surgical instruments. Similarly, example images from Sinus-cadaver and CholecSeg8k are shown in Figure 1b,c, respectively. Sinus-cadaver is a binary-class dataset collected from endoscopic surgery. As illustrated by the sample images, UW-Sinus-cadaver exhibits large variations in illumination, along with challenges such as blur, glare, low contrast, and size variability. CholecSeg8k contains multi-class segmentation masks and is considered one of the most challenging datasets for surgical instrument segmentation. Dataset details are provided in Section 3.1.

The rise of artificial intelligence is transforming and automating multiple sectors, including healthcare [8,9]. Deep learning is widely applied to disease diagnosis and assessment tasks, and has gained prominence by enabling effective early diagnosis and disease quantification. Extensive research is being conducted at the intersection of robotics and medicine [10]. Medical robotics is shaping the future of healthcare and is expected to facilitate broader adoption and commercialization of robot-assisted minimally invasive surgery.

Surgical instrument segmentation remains an active research area due to the increasing adoption of robotic surgery. Existing research has explored both deep feature-based and handcrafted feature-based approaches for surgical instrument segmentation. A residual recurrent method proposed by Yang et al. [6] employs densely connected attention mechanisms within a recurrent network to improve segmentation performance. This approach adopts U-Net as its backbone, which requires a large number of trainable parameters. Similarly, another study [7] applies several state-of-the-art segmentation architectures to segment anatomical structures, including surgical instruments, during laparoscopic procedures. In that study, segmentation networks were evaluated on the challenging CholecSeg8k dataset, where U-Net++ [11] reportedly outperformed other methods due to its semantic gap reduction capability. However, U-Net++ also exhibits computational limitations and requires a substantial number of parameters for training.

Another deep learning-based method [12] employs multiscale feature aggregation and retention mechanisms to enhance segmentation performance for colorectal polyps and surgical instruments. That work proposes two architectures—a base network and a final network—to combine low-level and high-level information through multiscale retention, thereby improving segmentation accuracy. The method requires 4.97 million trainable parameters, indicating satisfactory computational performance; however, the authors acknowledge room for further improvement in computational efficiency.

A recent research work, namely adaptive SAM (Segment Anything), presented by Paranjape et al. [13], extends SAM to improve performance, particularly on medical data. AdaptiveSAM introduces adaptable modifications to enable fast adaptability and prompt-based segmentation. However, AdaptiveSAM does not perform well on the surgical instrument segmentation task. Similarly, a recent study by Peng et al. [14]. improves training by generating and incorporating synthetic images. The combination of active learning with synthetic images and blending and fusion strategies results in improved segmentation accuracy; however, this approach is limited to binary segmentation.

Lin et al. [15] employ a generative adversarial network and image-to-image translation to reduce dependency on labeled data. They also introduce a customized loss function and evaluate their approach on surgical instrument segmentation from endoscopic images. Semantic inconsistency remains a minor limitation of this work. Another recent study by Jamal et al. [16]. proposes a framework called SurgDepth that leverages a multimodal strategy using both RGB and depth information. SurgDepth employs a vision transformer as the encoder and is evaluated on multiple surgical instrument datasets. Although SurgDepth demonstrates strong performance, its computational requirements, with 101.3 million trainable parameters, represent a notable limitation. Similarly, Grammatikopoulou et al. [17]. propose a neural architecture search-based model to optimize spatial and temporal processing for surgical segmentation. While this method achieves satisfactory segmentation performance, it requires a large number of trainable parameters (39.0 million).

We have divided previous studies into edge-aware and boundary-guided methods and briefly discussed them as follows.

Boundary-guided methods: Xu et al. [18] presented a boundary guidance network for segmenting medical images. They primarily used a boundary extraction module to guide the decoding process. FFMS-Net uses frozen Sobel/Laplacian priors instead of a learned boundary extraction/guidance subnetwork. One more research work presented by Wang et al. [19] introduced a boundary context neural network for the segmentation of medical images. During each encoding stage, they use pyramid edge extraction and explicit boundary/context coupling. FFMS-Net is lighter and avoids explicit boundary branches. Similarly, another research work by Zhang et al. [20] uses boundary enhancements with the help of an edge detector for the semantic segmentation. However, unlike the proposed method, they also apply the edge detection throughout the encoder, and edge maps are not preserved using multiple frozen filters. Moreover, this work employs the U-Net as the baseline network, while the FFMS-Net does not rely on any other base network.

Edge-aware methods: In a study, Bui et al. [21] presented a segmentation architecture with multiscale edge-guided attention. They explicitly explored the use of the Laplacian operator to preserve high-frequency edge information. Moreover, edge-guided attention is directly distributed to every stage of the decoder, making it computationally expensive (44.19 million parameters). FFMS-Net uses frozen Sobel + Laplacian priors in a frozen and learnable pipeline setting, without a separate edge-attention stack.

Another work presented by Peng et al. [22] uses mainstream edge-guided information with a collaborative module throughout the encoder. Contrary to FFMS-Net, in this architecture, edge features are extracted from all of the encoder stages and fed to the decoder stage. Overall, this is a computationally heavy architecture that requires 149.5 million parameters.

The proposed efficient design is specifically motivated by endoscopic artifacts where boundary cues are present but easily attenuated by early downsampling. While multi-scale and dilated features-based modeling have already been explored, the design of modules such as tri-atrous blending, frozen and learnable feature pipeline, and the progressively structure-preserving decoder itself, and especially the combination of these modules, is novel. Ablation studies in this research also confirm the reinforcement effect of each module and the joint production of a reliable performance. According to the best of our knowledge, none of the FFMS-Net main modules are a known block/module from any other architecture. The detailed novelty is presented in the contribution at the end of Section 1.

Researchers widely acknowledge the importance and scope of surgical instrument segmentation for enabling a smooth transition toward robot-assisted minimally invasive surgery [6,7]. Numerous state-of-the-art methods have been developed and evaluated for accurate surgical instrument segmentation. However, maintaining satisfactory segmentation performance under challenging imaging conditions remains difficult. Many existing approaches fail to deliver acceptable segmentation accuracy on challenging surgical data samples. Live endoscopic surgical procedures, characterized by a limited field of view, demand robust methods capable of handling unavoidable adverse imaging conditions. From a surgical perspective, accurate preservation of instrument boundaries and structural integrity is particularly important. Although achieving perfectly accurate boundary segmentation is unrealistic in challenging live surgical environments, attaining reliable performance for both structure and boundaries is highly desirable. Nevertheless, previous methods exhibit limitations in delivering consistent segmentation performance, particularly when predicting instrument boundaries and structural details.

Surgical datasets are often highly diverse, exhibiting substantial inter-dataset variability. Many existing methods rely on evaluation using a single dataset, which restricts the assessment of generalizability. Several existing approaches require a large number of trainable parameters, limiting their practical applicability. To address these challenges, we developed an advanced segmentation architecture with effective building blocks designed to perform robustly under challenging surgical conditions while requiring a very small number of trainable parameters (only 1.5 million).

The key contributions of the proposed research are summarized as follows.

- In this research work, an advanced segmentation architecture, namely the frozen-filters-based morphology-aware segmentation network (FFMS-Net), is developed to achieve reliable surgical instrument segmentation from endoscopic images. FFMS-Net introduces a frozen and learnable feature pipeline (FLFP) that simultaneously exploits frozen maps and learnable initial spatial features. The frozen component of FLFP, referred to as the frozen filter unit (FFU), is constructed using multiple frozen Sobel and Laplacian filters to preserve edge and structural information of the instruments, which is subsequently fused with learnable spatial features.
- A tri-atrous blending (TAB) block is incorporated at the end of the encoding to process and fuse multi-receptive-field-based contextual information, enabling the network to preserve surgical instrument morphology. The multi-rate dilated context in TAB allows the architecture to retain contextual and morphological details even under challenging conditions such as blur, blood obscuration, and smoke.
- A progressively structure-preserving decoder (PSPD) enables the model to learn and maintain the structural integrity of surgical instruments. In PSPD, features from different encoder stages are fused after each upsampling step, followed by aggregation of mildly dilated features. FFMS-Net is evaluated on three challenging datasets and outperforms state-of-the-art methods while requiring only 1.5 million trainable parameters.

The remainder of the paper is organized as follows. Section 2 and Section 3 present the proposed methodology and experimental results, respectively. Section 4 discusses the findings, and Section 5 concludes the study.

## 2. Materials and Methods

### 2.1. Databases

FFMS-Net is evaluated using three challenging open datasets of endoscopic UW-Sinus-surgery-live [6], UW-Sinus-surgery-cadaver [6], and CholecSeg8k [7]. Sample images from all three datasets are shown in Figure 1. UW-Sinus-surgery-live contains 4658 images with pixel-level surgical instrument annotations. Images were resized to 256 × 256 pixels for training. This dataset includes numerous cases with surgical instruments affected by blur, blood occlusion, smoke, low contrast, glare, and small object size. Similarly, the UW-Sinus-surgery-cadaver dataset contains 4345 images, which were resized to 240 × 240 pixels for network training. This dataset presents comparable challenges, including large illumination variations, severe glare, and metallic reflections. Both datasets were created by the Biorobotics Lab at the University of Washington, Seattle, WA, USA. The CholecSeg8K dataset contains 8080 images acquired from 17 laparoscopic video procedures. This dataset includes 13 classes; however, several classes are severely underrepresented. Following state-of-the-art practice, these classes were merged into a single class termed “Misc [7].” As shown in Figure 1, CholecSeg8k exhibits a pronounced domain gap across images from different procedures, making segmentation particularly challenging.

### 2.2. Methodology

#### 2.2.1. Summary of Proposed Method

**Figure 2:**
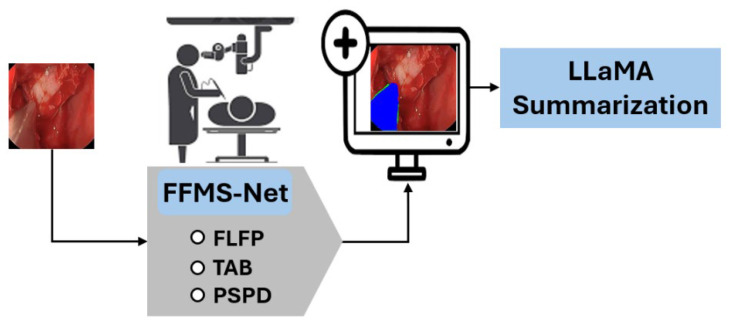
The overview of the proposed methodology.

An overview of the proposed methodology is illustrated in Figure 2. Input images from the training split are provided to FFMS-Net to obtain a trained model. The trained network is subsequently applied to unseen test images to generate prediction masks. Accurate surgical instrument segmentation is essential for enhancing robot-assisted minimally invasive surgery. In live surgical settings with a limited field of view, preserving edge information and morphological structure is critical for achieving reliable segmentation accuracy. FFMS-Net employs the FLFP, TAB, and light skip paths (LSPs) during encoding to maintain robust performance under challenging imaging conditions. In addition, the PSPD is used to retain structural information of the target classes throughout decoding. The detailed architecture and operational flow of FFMS-Net are described in the subsequent subsection.

As shown in the overview diagram, pixels corresponding to surgical instruments are accurately identified and separated from the background. Based on the resulting prediction mask, deterministic descriptors are computed, and a non-clinical textual summarization of the surgical scene is generated using the open-source large language model Meta AI (LLaMA) [23]. The proposed method is trained independently from scratch and does not rely on weight migration or pretrained models.

#### 2.2.2. Network Diagram of FFMS-Net and Its Function

Surgical instrument segmentation is essential for automating or intelligently assisting surgical procedures. As discussed earlier, challenging conditions during live surgery, such as blur, glare, low contrast, blood occlusion, smoke, and variable illumination, make segmentation particularly difficult. In addition, a robust framework is desirable that can deliver accurate predictions without demanding excessive numbers of trainable parameters. To address these challenges, FFMS-Net is developed to achieve reliable segmentation performance while requiring a very small number of trainable parameters (only 1.5 million) compared with existing methods.

**Figure 3:**
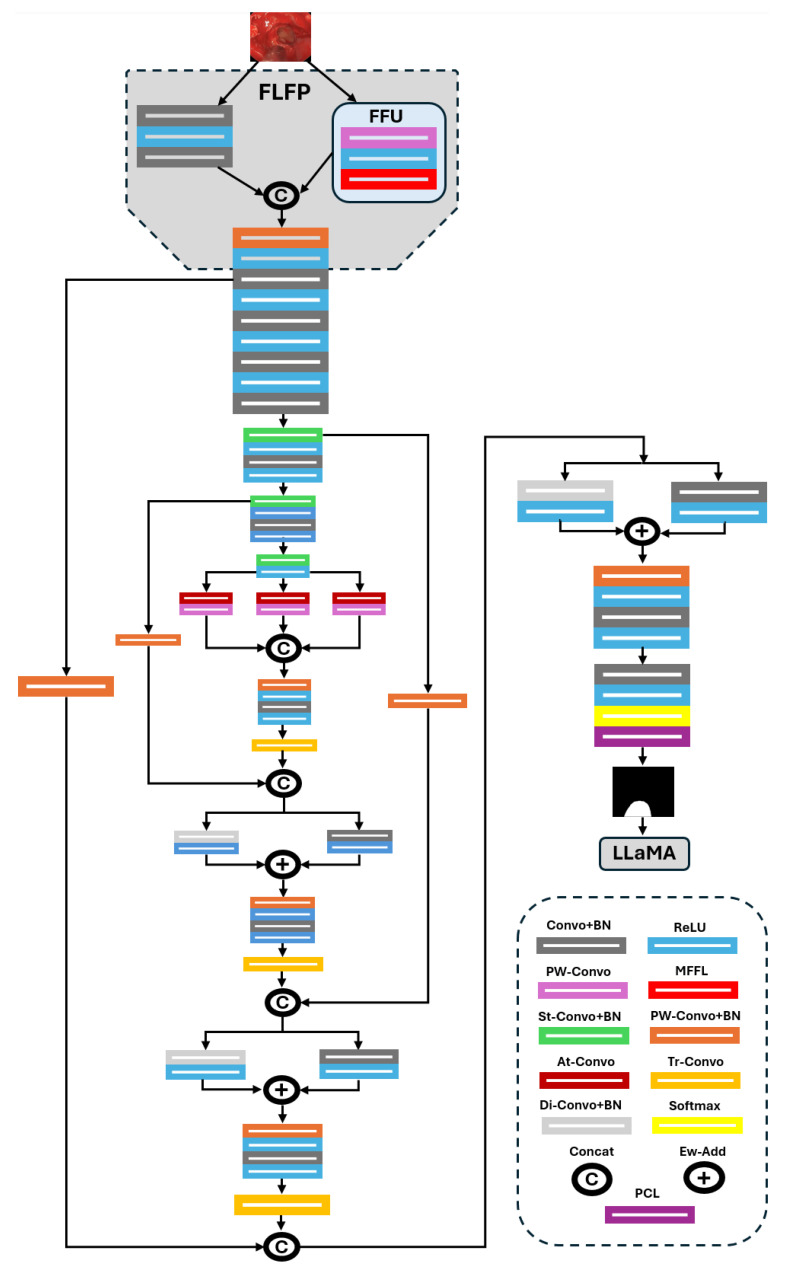
Architecture of FFMS-Net for surgical instrument segmentation. (FLFP: frozen and learnable pipeline; FFU: frozen filter unit; Convo: convolutional layer; BN: batch normalization layer; St-Convo: strided convolutional layer; MFFL: multi-filter-based frozen layer; Tr-Convo: transposed convolutional layer; PW-Convo: point-wise convolutional layer; At-Convo: atrous convolutional layer; Di-Convo: dilated convolutional layer; Concat: depth-wise concatenation; Ew-Add: element-wise addition; PCL: pixel classification layer).

The network diagram of FFMS-Net is shown in Figure 3. FFMS-Net is designed as a standalone architecture and does not use any existing network as a backbone. As illustrated in the diagram, FFMS-Net incorporates several effective building blocks, including the FLFP, TAB, PSPD, and connective LSPs, which collectively contribute to robust performance. Input images are fed into FFMS-Net through the image input layer. A key challenge in surgical instrument segmentation is accurate detection of instrument edges and morphology, particularly under adverse imaging conditions. FFMS-Net addresses this challenge using an FLFP stem that processes, learns, and fuses frozen maps with learnable spatial features. Within FLFP, features extracted from the input image are split into learnable and frozen branches. In the FFU, input features are first passed through a point-wise convolution (PW-convo) followed by a rectified linear unit (ReLU), and then fed into a multi-filter-based frozen layer (MFFL). The MFFL consists of three frozen filters initialized using Sobel and Laplacian operators to preserve edge maps and orientation information of surgical instruments. The resulting frozen maps are fused with features processed through a set of standard convolutional layers. The fused features are subsequently squeezed using PW-convo, batch normalization (BN), and ReLU before being passed to the encoder.

These squeezed features are further processed using convolutional and BN layers and are transferred through LSP to be fused at C4 near the end of decoding. Features from FLFP are then propagated through a sequence of convolution, BN, and ReLU layers to enhance feature activation. Spatial resolution is progressively reduced using strided convolutional layers (St-convo), each with a stride of 2. Between strided convolutions, features are further refined using convolution, BN, and ReLU operations. The final strided convolution produces the lowest-resolution feature representation, which is subsequently forwarded to the TAB block.

As discussed earlier, surgical instrument datasets commonly include images in which instrument visibility is degraded by blur, blood occlusion, smoke, and morphological variations. Preserving morphological continuity of surgical instruments under such challenging conditions is particularly difficult. The TAB block processes multi-receptive-field contextual information to better address these challenges while maintaining morphological continuity [24]. TAB consists of three parallel atrous feature streams with different receptive fields, each followed by a point-wise convolution (PW-convo), after which all parallel features are fused. This multi-receptive-field contextual modeling enables TAB to capture long-range morphological continuity, especially under challenging imaging conditions.

The fused features from TAB are subsequently passed to the first transposed convolution (Tr-convo) layer of the PSPD after further activation through convolution, BN, and ReLU layers. Surgical instrument datasets collected during live procedures exhibit substantial structural variability. Moreover, during upsampling, small or poorly visible instruments are particularly susceptible to spatial information loss. PSPD is therefore designed to progressively preserve instrument structure throughout decoding.

Features upsampled by the first Tr-convo are fused with high-level encoder features at C2 via LSP. Each LSP consists of a PW-convo followed by BN. As illustrated in Figure 1, surgical instrument segmentation is often characterized by severe class imbalance, and LSPs help suppress background-dominated channels. In addition, LSPs reduce the semantic gap between encoder and decoder features, enabling improved performance for small and thin surgical instruments.

The fused features at C2 are then split into two parallel paths: one employing a slightly dilated convolution (Di-convo) and the other using a standard convolution. Standard convolutions are effective for detecting sharp edges, whereas slightly dilated convolutions help avoid over-smoothing and assist in detecting blurred or blood-occluded instruments [25]. The outputs of these parallel paths are aggregated using element-wise addition at A1. Similarly, after each upsampling operation, multi-scale spatial information from the encoder is fused with upsampled decoder features via LSPs. This progressive multi-scale feature fusion further supports structural preservation, even under challenging conditions such as glare or metallic reflections. At C4, features from the FLFP section are fused with the final upsampled features, and the resulting split features are aggregated at A3. These aggregated features are processed through convolution, BN, and ReLU layers, followed by a softmax layer, and are finally passed to the pixel classification layer (PCL) to generate the prediction mask. Deterministic descriptors are computed from the prediction mask. Subsequently, these descriptors, together with the original images and predicted masks, are provided to the open-source LLaMA-3 [23] to generate a simple non-clinical summarization (details of LLaMA are discussed in the Section 4).

#### 2.2.3. Experimental Details and Data Preparation

FFMS-Net is evaluated using three challenging open datasets—UW-Sinus-surgery-live, UW-Sinus-surgery-cadaver, and CholecSeg8k—collected from endoscopic and laparoscopic surgeries, respectively. Following the state-of-the-art methods, for a fair comparison, no data augmentation was applied, as sufficient data were available for training especially for UW-Sinus-live and UW-Simus-Cadaver datasets. No weight migration or backbone architectures were employed; the network is trained from scratch. Network design and experimental evaluation were conducted using the Matlab 2025a framework [26] on an Intel i9-14900K system with 64 GB RAM and an NVIDIA GeForce RTX 4090 GPU [27].

**Figure 4:**
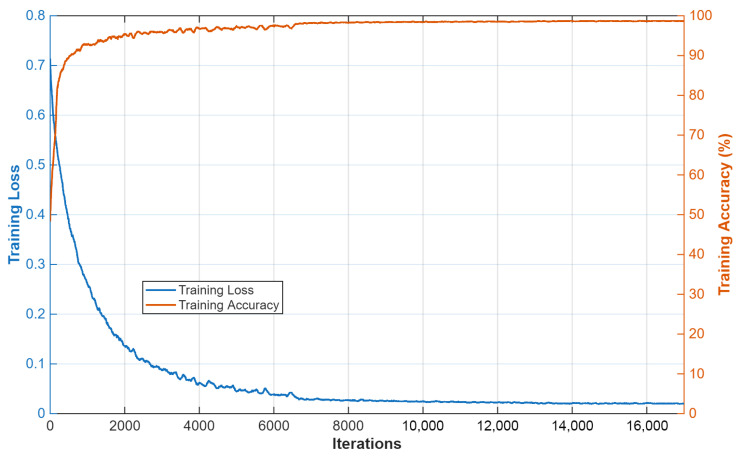
FFMS-Net training loss and accuracy plot.

Images from UW-Sinus-surgery-live and UW-Sinus-surgery-cadaver were resized to 256 × 256 and 240 × 240 pixels, respectively, for training. All experiments were conducted with default random initialization settings. No fixed random seed was enforced. Because of the lightweight design of the proposed method, the training time for most of the training was 25–35 min. Both datasets involve binary classification, and the same data splitting and evaluation protocol (five-fold cross-validation) described in [6,12] was followed to ensure fair comparison. The CholecSeg8k dataset includes 13 classes, several of which are severely underrepresented; following prior work [7], these classes were merged into a single “Misc” class. The same data distribution and evaluation protocol reported in [7] was adopted. Images from CholecSeg8k were resized to 512 × 512 pixels for training. Although multi-class segmentation results are reported for CholecSeg8k, the primary class of interest remains surgical instruments, consistent with the study scope. Accordingly, quantitative class-wise results are presented only for the surgical instrument class. Weighted Dice loss [28] was used for CholecSeg8k to address multi-class supervision and severe class imbalance, whereas weighted cross-entropy loss [29] was employed for both sinus datasets due to their binary nature and comparatively lower class imbalance. All experiments were conducted under fixed training schedules, including optimizer choice, learning rate, batch size, regularization, augmentation policy, and epoch budget. Adam [30] was used as the optimization algorithm in all experiments due to its favorable convergence behavior. The initial learning rate, step decay, and L2 regularization were set to 0.0005, 10 by 0.1, and 0.0005, respectively. Mini-batch sizes of 10 and 14 were used for CholecSeg8k and the sinus datasets, respectively. The CholecSeg8k model was trained for 26 epochs, whereas the sinus datasets were trained using five-fold cross-validation with fold-specific epoch schedules depending on the training partition size. Training data were shuffled at every epoch. Default pseudorandom configuration of MATLAB [26] was used, and no explicit fixed random seed was enforced during training; therefore, minor variations due to random initialization and shuffled mini-batches may occur. However, in informal checks with repeated training on a subset of the data, the variation in instrument dice similarity coefficient was within approximately ±0.3 percentage points, suggesting that the optimization is relatively stable. Moreover, to reduce dependence on a single partition, all results of both main UW-sinus datasets were reported as averages across five folds. In future work, controlled random-seed experiments and repeated runs will be conducted to further quantify run-to-run variance (noted as future work in Section 4.3). The training accuracy and loss curves of FFMS-Net on CholecSeg8k are shown in Figure 4.

## 3. Experimental Results

### 3.1. Evaluation Metrics

The proposed FFMS-Net is evaluated using three datasets. The network is independently trained from scratch and applied to unseen test sets. The trained FFMS-Net model is used to generate prediction masks for unseen test images to evaluate the proposed method. UW-Sinus-Surgery-Live and UW-Sinus-Surgery-cadaver are binary-class datasets, whereas CholecSeg8K is a multi-class dataset. For each dataset, the trained network is applied to the corresponding test set, and prediction masks are generated to compute both quantitative and qualitative results. For semantic segmentation, the Dice similarity coefficient (D) and Intersection over union (I) are widely accepted evaluation metrics [31,32]. The mathematical expressions of these measures are defined as follows. Results are computed based on true negative (tn), false positive (fp), true positive (tp), and false negative (fn) pixels.

(1) $$I=\frac{tp}{tp+fp+fn}$$

(2) $$D=\frac{2\;tp}{2\;tp+fp+fn}$$

### 3.2. Results of FFMS-Net on UW-Sinus-Surgery-Live Dataset

Both quantitative and qualitative results obtained using the UW-Sinus-Surgery-Live dataset are presented in this subsection. Five-fold cross-validation results are reported in Table 1 to enable fair comparison with state-of-the-art methods. The quantitative results indicate that FFMS-Net achieves reliable performance despite the challenging nature of the UW-Sinus-Surgery-Live dataset. The ablation study with the UW-Sinus-Surgery-Live dataset is presented in Table 2. Segmentation accuracies presented in Table 2 confirm the effectiveness of all main modules of the FFMS-Net. Moreover, the performance achieved by FFMS-Net is compared with previous methods in Table 3. The comparative quantitative analysis demonstrates that FFMS-Net outperforms existing approaches, attributable to its effective architectural building blocks, while requiring only 1.5 million trainable parameters. All reported results are expressed as percentages. Lastly, cross-dataset evaluation is presented in Table 4. In cross-dataset evaluation, the trained model of the UW-Sinus-Surgery-Cadaveric dataset was tested with the UW-Sinus-Surgery-Live dataset. For each fold *k*, the model trained on the Sinus-Cadaveric dataset fold *k* was evaluated on the corresponding Sinus-live fold *k* test subset. The performance drop in semantic segmentation is common in cross-dataset evaluation. As shown in the sample images of the datasets, both datasets exhibit substantial differences in domain, illumination, tissue dynamics, blood occlusions, specular reflections, blur, and color distributions. Despite a significant domain gap during cross-dataset evaluation, FFMS-Net still manages to maintain almost half of the single-dataset-based performance. Special augmentation schemes designed to overcome the domain gap might help in this case, and it is noted as one of the future directions of this study (details about performance drop for cross-dataset evaluation are included in Section 4.3).

#### Sample Qualitative Results Produced by FFMS-Net with UW-Sinus-Surgery-Live Dataset

The UW-Sinus-Surgery-Live dataset is among the most challenging datasets for surgical instrument segmentation, as it is collected during live endoscopic surgery. The dataset includes numerous difficult cases characterized by blur, smoke, small instrument size, and low contrast. Nevertheless, FFMS-Net addresses these challenges through the effective integration of architectural components such as FLFP, TAB, and PSPD.

**Figure 5:**
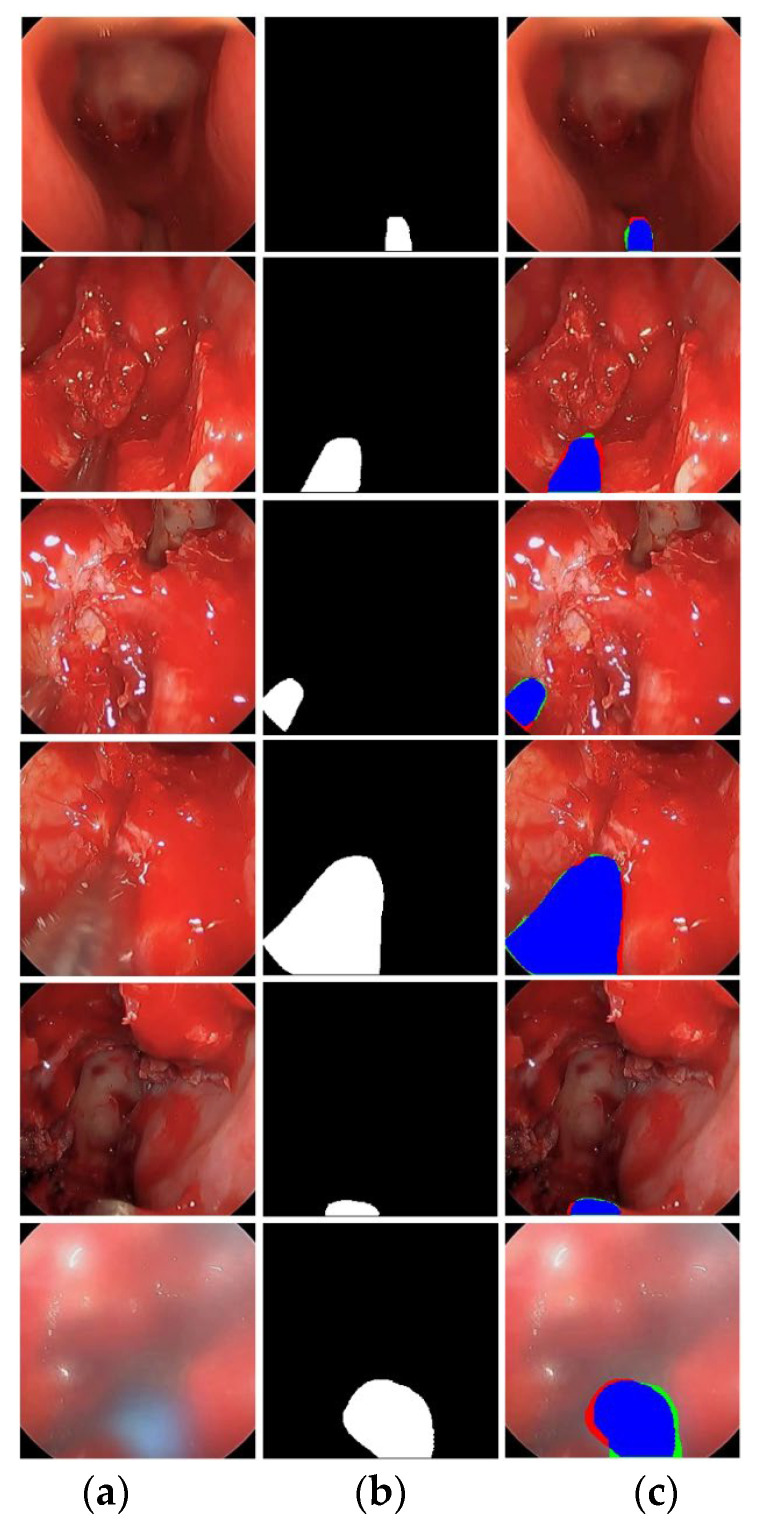
Qualitative examples of good and relatively poor segmentation results produced by FFMS-Net on the UW-Sinus-Surgery-Live dataset. (**a**) Testing image, (**b**) ground-truth image, and (**c**) segmented image (tp pixels: blue; fp pixels: green; fn pixels: red).

As shown in Figure 5, representative examples of good segmentation results (first five rows) and relatively poor segmentation result (last row) are presented. The qualitative results demonstrate that FFMS-Net maintains reliable segmentation performance even under challenging imaging conditions. The relatively poor segmentation observed in the last row is primarily attributed to extreme blur; however, FFMS-Net is still able to identify plausible instrument pixels.

### 3.3. Results of FFMS-Net on UW-Sinus-Surgery-Cadaver Dataset

The evaluation of FFMS-Net is further extended to the UW-Sinus-Surgery-Cadaver dataset. The characteristics of this dataset differ from those of the dataset discussed in the previous subsection. It exhibits substantial illumination variation, along with additional challenges such as glare, low contrast, small instrument size, and metallic reflections. Despite these challenges, FFMS-Net maintains reliable performance due to the architectural components designed to handle adverse imaging conditions. Table 5 reports the five-fold cross-validation results obtained on the UW-Sinus-Surgery-Cadaver dataset. In addition to the dice similarity coefficient and Intersection over union, we also expanded the evaluation using precision, recall, and boundary F1 (BFScore), which evaluates boundary alignment with a tolerance. Furthermore, the performance achieved by FFMS-Net is compared with state-of-the-art methods in Table 6. The comparative results demonstrate that FFMS-Net achieves superior performance while requiring a minimal number of trainable parameters.

#### Sample Qualitative Results Produced by FFMS-Net with UW-Sinus-Surgery-Cadaver Dataset

**Figure 6:**
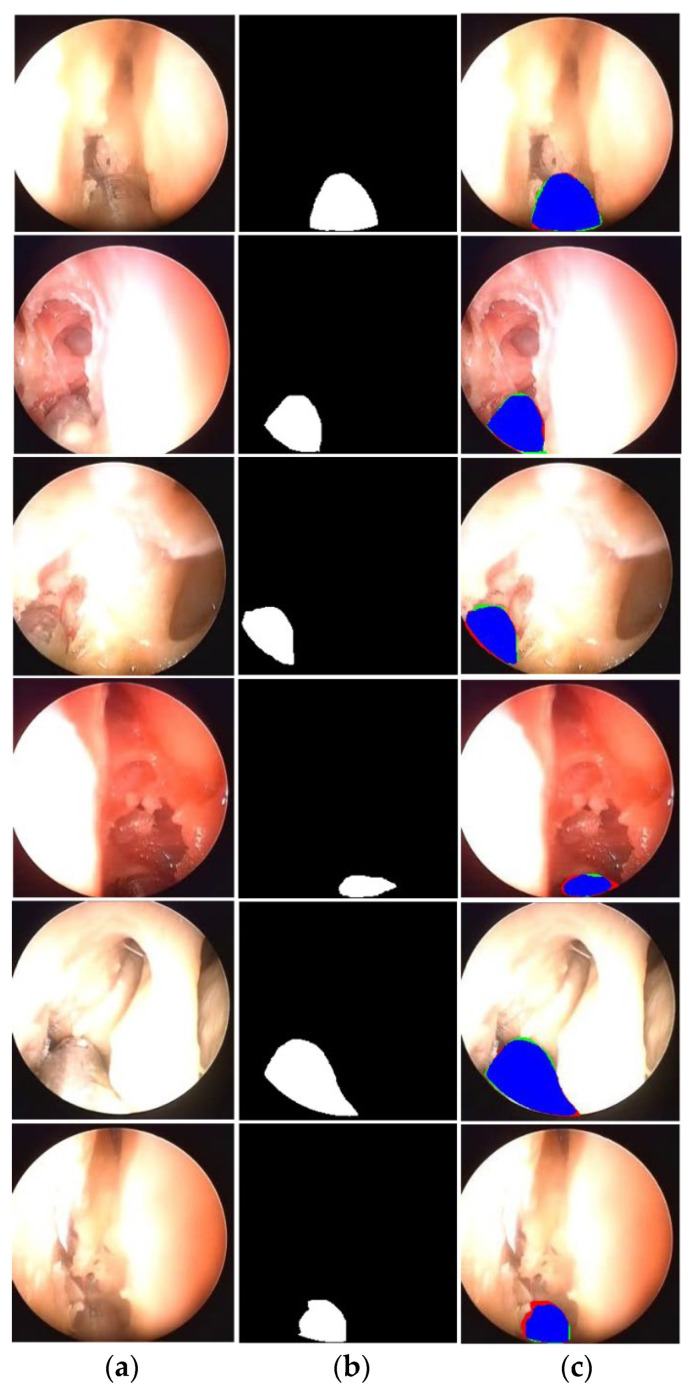
Qualitative examples of good and relatively poor segmentation results produced by FFMS-Net on the UW-Sinus-Surgery-Cadaver dataset. (**a**) Testing image, (**b**) ground-truth image, and (**c**) segmented image (tp pixels: blue; fp pixels: green; fn pixels: red).

The qualitative results generated by FFMS-Net are presented in Figure 6. The example images pose significant challenges to segmentation due to illumination variation, blur, glare, low contrast, and small instrument size in several cases. Nevertheless, FFMS-Net maintains reliable performance despite these adverse visual conditions. Representative examples of good segmentation results are shown in rows 1–5, whereas relatively poor segmentation results are illustrated in the last row. The relatively poor performance observed in the last row is primarily attributable to exceptionally difficult imaging conditions; however, FFMS-Net still produces plausible segmentation outputs.

### 3.4. Results of FFMS-Net on CholecSeg8K Dataset Along with Ablation Studies

The CholecSeg8K dataset is considered among the most challenging datasets owing to the pronounced domain gap, particularly for the data split adopted in [7]. Both qualitative and quantitative results reported in [7] confirm the difficulty of this task. To further validate the effectiveness of FFMS-Net, the proposed method is also evaluated on the CholecSeg8K dataset. As described in Section 2.2.3, experiments are conducted using multi-class segmentation; however, in line with the scope of this study, the primary focus remains on the surgical instrument class. Accordingly, multi-class qualitative results are presented for visual interpretation, whereas class-wise quantitative results are reported only for the surgical instrument class. In addition, overall quantitative results across all classes are provided for comparison with existing methods.

Table 7 summarizes the results of the ablation studies. These results confirm the contribution of each architectural component to the overall performance. Since LSPs operate in conjunction with the PSPD, they are evaluated jointly in the ablation analysis. Complete removal of PSPD is not feasible owing to its role in generating prediction masks; however, in the first ablation setting, all fusion and aggregation operations within PSPD are removed to assess their impact on performance.

The results achieved by FFMS-Net are compared with existing methods in Table 8. Despite the substantial domain gap and other challenging imaging conditions, FFMS-Net demonstrates reliable performance for surgical instruments as well as for all classes. While many existing approaches require a large number of trainable parameters, FFMS-Net achieves this performance with only 1.5 million trainable parameters. Moreover, no data augmentation was intuitively applied, as sufficient data were available for training. Still, extra experiments with basic augmentation (flipping, rotation, cropping, etc.) only on the training data were conducted. The result for the segmentation of the instrument was almost the same as without augmentation. The thin structure of the surgical instrument and sufficient training data availability could be the reason that augmentation could not contribute to a significant performance difference. However, advanced and dedicated augmentation schemes designed to overcome the domain gap might help in this case, and it is noted as one of the future directions of this study (details about future work are provided in Section 4.3).

#### Qualitative Results Produced by FFMS-Net with the CholecSeg8K Dataset

**Figure 7:**
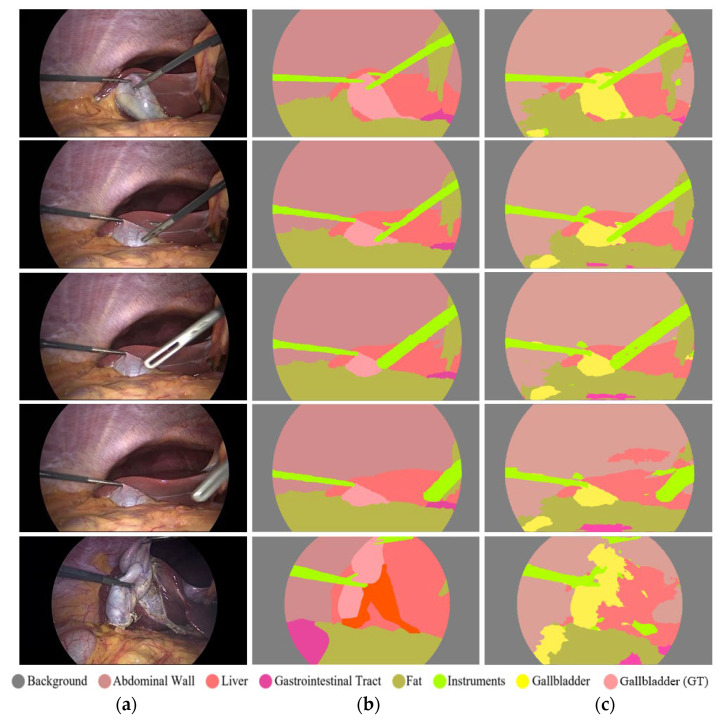
Good and relatively poor qualitative results for segmentation using FFMS-Net on CholecSeg8K dataset. (**a**) Testing image, (**b**) ground truth image, and (**c**) segmented image.

As described in Section 2, CholecSeg8K is among the most challenging datasets due to extensive variations, including a pronounced domain gap across different surgical clips. CholecSeg8K is a multi-class dataset; multi-class segmentation results are presented for visual interpretation, whereas surgical instrument segmentation remains the primary focus of this study. As shown in Figure 7, examples of acceptable segmentation results are illustrated in rows 1–4, while a relatively poor segmentation example is shown in the last row. The same colors are used in both the segmented image and the ground truth image, except for the gallbladder, which is shown in yellow in the segmented image. FFMS-Net demonstrates reliable performance, particularly for surgical instrument segmentation. Although color labels for other classes are visualized and FFMS-Net achieves acceptable overall performance, the poor result in the last row highlights performance limitations. Such degradation is not unique to FFMS-Net; similar performance degradation has been reported for state-of-the-art methods on CholecSeg8k under comparable conditions because of the severe domain gap [7].

## 4. Discussion

The integration of artificial intelligence and robotics is transforming multiple sectors, including healthcare. Consequently, robot-assisted minimally invasive surgery has attracted substantial research interest. Surgical instruments are essential components of any surgical procedure; however, live endoscopic surgeries introduce numerous limitations and challenges for pixel-level detection of surgical instruments [5]. The visual challenges associated with accurate surgical instrument segmentation have been discussed in previous sections. In addition to achieving high segmentation performance under challenging imaging conditions, computational efficiency, particularly the number of required trainable parameters, is also an important consideration. Table 9 compares the number of trainable parameters required by FFMS-Net with those of existing methods. FFMS-Net requires only 1.5 million trainable parameters, which is substantially lower than that of other approaches. Moreover, to check hardware efficiency, we computed inference throughput on an NVIDIA GeForce RTX 4090 GPU. On the UW-Sinus-Surgery-Live dataset, the proposed model required an average of 0.0129 s per image, corresponding to 77.56 frames per second (FPS). The throughput of FFMS-Net is more than the typical surgical video frame rates (25–30 FPS). This shows that the model is computationally efficient and suitable for near-real-time or real-time surgical instrument segmentation.

From a translation perspective, dependable surgical instrument segmentation can support multiple sub-functions in robotic systems and surgical workflows. The predictions masks of the instrument can enable tool tracking, motion analysis, definition of collision-sensitive regions, and contextual analysis of surgical video streams. FFMS-Net’s compact design (1.5 million parameters) is advantageous for deployment in resource-constrained environments. However, for clinical application, prospective validation, robustness evaluation under severe occlusion, and careful safety analysis before clinical decision support or robotic platform integration are required.

### 4.1. Proof-of-Concept: Non-Clinical LLM-Based Scene Summarization

**Figure 8:**
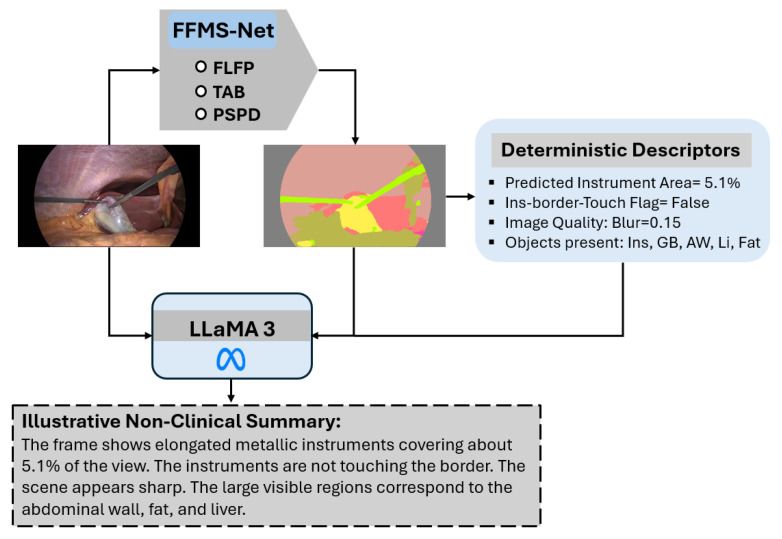
Proof-of-concept illustration of non-clinical LLaMA-based scene summarization from FFMS-Net outputs (Ins: Instrument; GB: Gallbladder, AW: Abdominal wall; Li: Liver).

As shown in Figure 8, a lightweight LLM-based [23] scene summarization module is included to illustrate one possible downstream application of the predicted masks. A small set of deterministic descriptors is computed from the prediction mask, including instrument area ratio, border-touch status, class presence, and a blur-related image quality indicator. These descriptors, together with the image and predicted mask, are provided to open-source LLaMA-3 to generate a short natural-language description of the scene. This component is included only as an exploratory proof-of-concept. The generated summaries are intended only for non-clinical interpretation; no expert validation, user study, or clinical decision-making assessment was performed in this work. The summary-quality evaluation and expert validation are explicitly noted as future work in Section 4.3.

### 4.2. Visual Demonstration of FFMS-Net Focusing Instrument Features Using Grad-CAM

**Figure 9:**
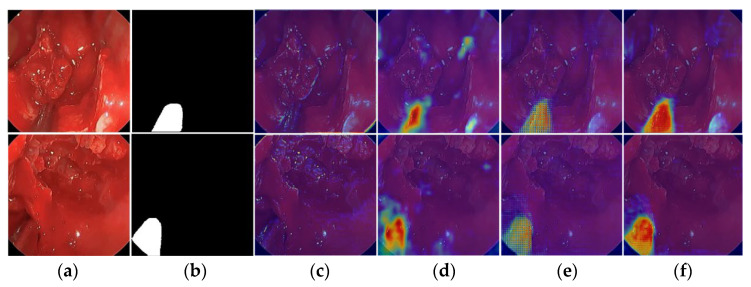
Visualization of feature learning in FFMS-Net on the UW-Sinus-Surgery-Live dataset using Grad-CAM heat activation maps. (**a**) Original image, (**b**) ground-truth image, and (**c**–**f**) heat activation maps extracted from different stages of FFMS-Net.

To demonstrate the progressive learning of meaningful features by FFMS-Net, heat activation maps are extracted using Grad-CAM [42]. At early stages, the network attends to broader anatomical structures; at deeper stages, it increasingly focuses on features of the target class (surgical instrument). As shown in Figure 9, Grad-CAM visualizations are extracted from different stages of FFMS-Net using challenging samples from the UW-Sinus-Surgery-Live dataset. In the deeper layers, particularly in Figure 9d,e, FFMS-Net more clearly delineates the most relevant instrument features. As illustrated in Figure 9f, FFMS-Net accurately concentrates on the spatial features and morphology of surgical instruments, even under low-contrast, blurred, and blood-occluded conditions. These mainly considered features of the surgical instruments by FFMS-Net are represented by Grad-CAM with reddish-orange color in Figure 9f.

### 4.3. Limitations and Future Work

Despite the encouraging results, several limitations need to be mentioned. First, despite cross-dataset experimental results, domain shift between surgical environments remains a challenging task. Future research could explore more robust domain adaptation or augmentation strategies specifically designed to handle cross-domain evaluation. Second, the LLM-based scene summarization component was presented only as a proof-of-concept and has not been evaluated through user studies or clinical validation. Third, this study primarily focused on segmentation accuracy and computational efficiency; its clinical applicability could be further strengthened through extensive validation. These directions present important tasks for future research. Lastly, controlled random-seed experiments and repeated runs will be conducted to further quantify run-to-run variance in the future.

In Table 4, cross-dataset evaluation results showed a significant performance degradation compared to testing with the same dataset, indicating that domain shift remains a major challenge. In the cases of the Sinus-live and Sinus-cadaver datasets, images exhibited distinct differences in tissue appearance, blood content, specular reflections, lighting stability, and motion-related artifacts, all of which impacted generalization. Although FFMS-Net maintained partial robustness under these shifts, the observed degradation suggests that morphology-aware design alone is insufficient for full cross-domain transfer. Therefore, future research should explore endoscope-specific data augmentation, domain adaptation, and domain generalization strategies to enhance robustness across the entire surgical environment.

## 5. Conclusions

Accurate surgical instrument segmentation is crucial for the advancement of robot-assisted minimally invasive surgery. Challenging imaging conditions during live procedures, such as blur, blood occlusion, low contrast, smoke, varying illumination, glare, and small instrument size, make reliable segmentation difficult. To address these challenges, we designed a robust architecture, FFMS-Net, to achieve reliable performance under adverse conditions. FFMS-Net employs an FLFP stem to process and fuse frozen and learnable features, preserving edge maps and structural information. In addition, a TAB block is incorporated at the end of the encoder to retain morphological and contextual information despite challenging imaging conditions.

Surgical instrument structures exhibit substantial variability; therefore, a progressively structure-preserving decoder is designed in which feature fusion followed by aggregation of slightly dilated features is performed after each upsampling step to maintain structural integrity. FFMS-Net is extensively evaluated on three datasets and demonstrates reliable performance while requiring only 1.5 million trainable parameters. Specifically, FFMS-Net achieves Dice scores of 89.30%, 93.02%, and 75.16% on the UW-Sinus-Surgery-Live, UW-Sinus-Surgery-Cadaver, and CholecSeg8k datasets, respectively, for surgical instrument segmentation. In the proposed framework, deterministic descriptors are computed from the prediction mask generated by FFMS-Net. The prediction mask, together with the original image and deterministic descriptors, is provided to an open-source LLaMA model for non-clinical natural-language summarization.

## Figures and Tables

**Table 1:** Five-fold cross-validation results of FFMS-Net using the UW-Sinus-Surgery-Live dataset (in %). (D: Dice similarity coefficient; I: Intersection over union; PRE: Precision; REC: Recall).

Folds	D	I	PRE	REC
Fold 1	90.39	83.81	90.66	91.68
Fold 2	90.86	84.85	88.44	90.36
Fold 3	89.46	83.33	88.25	92.28
Fold 4	88.81	82.32	87.93	91.66
Fold 5	86.98	79.87	87.16	89.72
Average	89.30	82.84	88.48	91.14

**Table 2:** Ablation studies for surgical instrument segmentation using the UW-Sinus-Surgery-Live dataset (in %). (D: Dice similarity coefficient; I: Intersection over union).

FLFP	TAB	PSPD + LSP	D	I
			79.55	71.81
√		√	81.6	73.45
		√	80.56	72.43
	√	√	82.18	74.38
√	√	√	89.30	82.84

**Table 3:** Comparing the results produced by FFMS-Net with existing methods on the UW-Sinus-Surgery-Live dataset (in %). (D: Dice similarity coefficient; I: Intersection over union).

Methods	D	I
TernausNet (VGG16) [33]	79.5	73.4
LWANet (MobileNet) [34]	72.6	65.2
DeepLabv3+ (ResNet50) [35]	81.8	75.2
DeepLabv3+ (ResNet50-b3) [35]	85.5	80.0
DeepLabv3+ (MobileNet-p8) [32]	83.2	77.0
MAFA-Net [36]	-	82.1
MAFA-Net [37]	87.7	82.1
DRR-Net [6]	88.26	78.99
MFRA-Net [12]	88.57	81.73
FFMS-Net (Proposed)	89.30	82.84

**Table 4:** Cross-dataset five-fold cross-validation results of FFMS-Net trained with the UW-Sinus-Surgery-Cadaver dataset and tested on the UW-Sinus-Surgery-Live dataset (in %). (D: Dice similarity coefficient).

Folds	D
Fold 1	52.34
Fold 2	41.79
Fold 3	37.07
Fold 4	47.5
Fold 5	37.92
Average	43.32

**Table 5:** Five-fold cross-validation results of FFMS-Net on the UW-Sinus-Surgery-Cadaver dataset (in %). (D: Dice similarity coefficient; I: Intersection over union; PRE: Precision; REC: Recall; BF1: Boundary F1).

Folds	D	I	PRE	REC	BF1
Fold 1	93.17	88.24	91.66	95.77	63.37
Fold 2	93.64	88.91	93.59	94.63	64.27
Fold 3	93.34	88.68	93.18	94.52	63.51
Fold 4	91.97	87.13	91.09	93.84	61.61
Fold 5	93.01	88.26	92.04	95.43	64.52
Average	93.02	88.24	92.31	94.84	63.45

**Table 6:** Comparing the results produced by FFMS-Net with existing methods on the UW-Sinus-Surgery-Cadaver dataset (in %). (D: Dice similarity coefficient; I: Intersection over union).

Methods	D	I
TernausNet (VGG16) [33]	85.4	80.1
LWANet (MobileNet) [34]	81.1	74.9
DeepLabv3+ (ResNet50) [35]	86.0	81.0
DeepLabv3+ (ResNet50-b3) [35]	88.9	84.0
DeepLabv3+ (MobileNet-p8) [35]	86.4	81.1
MAFA-Net [36]	90.2	85.6
MAFA-Net [37]	91.2	86.8
DRR-Net [6]	92.27	85.64
FFMS-Net (Proposed)	93.02	88.24

**Table 7:** Ablation studies for surgical instrument segmentation using the CholecSeg8K dataset (in %). (D: Dice similarity coefficient; I: Intersection over union).

FLFP	TAB	PSPD + LSP	D	I
			50.32	33.62
√		√	71.01	55.49
		√	61.43	44.33
	√	√	67.70	51.17
√	√	√	75.16	60.21

**Table 8:** Comparing the results produced by FFMS-Net with existing methods on the CholecSeg8K dataset (in %). (D: Dice similarity coefficient; I: Intersection over union; Ins: Instrument class; All classes: All classes including instrument).

Method	D		I	
	Ins	All Classes	Ins	All Classes
SAM-ZS [13]	2.5	-	2.5	-
Adaptive SAM [13]	45.0	-	39.0	-
U-Net [38]	52.0	54.0	-	43.0
DynUNet [39]	57.0	60.0	-	52.0
MedT [13]	59.5	-	50.0	-
U-Net++ [11]	61.0	62.0	-	55.0
DeepLabv3+ (MobileNet-p8) [35]	62.0	61.0	-	50.0
TransUNet [13]	62.5	-	56.0	-
UNetR [40]	71.0	60.0	-	49.0
FFMS-Net (Proposed) (with augmentation)	74.98	-	59.97	-
FFMS-Net (Proposed)	75.16	65.52	60.21	55.27

**Table 9:** Comparing computational requirements with existing methods for the UW-Sinus-Surgery-Live dataset (M: Million; FPS: Frames per second).

Methods	Parameters	Avg Inference Time/Image	FPS
TransUnet [40]	96.07 M	-	-
U-NetR [40]	92.58 M	-	-
U-Net [38]	31.04 M	-	-
U-Net++ [11]	9.04 M	-	-
DeepLab+V3+ [41]	20.6 M	-	-
MFRA-Net [12]	4.9 M	-	-
DynUnet [40]	19.07 M	-	-
FFMS-Net (proposed)	1.5 M	0.0129 s	77.56

## Data Availability

The original data presented in the study are openly available via the CholecSeg8k: https://www.kaggle.com/datasets/newslab/cholecseg8k accessed on 16 August 2025 and UW-Sinus-surgery-live and UW-Sinus-surgery-cadaver: https://github.com/SURA23/Sinus-Surgery-Endoscopic-Image-Datasets accessed on 2 August 2025. Code and trained models will be released after acceptance and upon reasonable request from the first author.
